# Mussels realize Weierstrassian Lévy walks as composite correlated random walks

**DOI:** 10.1038/srep04409

**Published:** 2014-03-18

**Authors:** Andy M. Reynolds

**Affiliations:** 1 Rothamsted Research, Harpenden, Hertfordshire, AL5 2JQ, UK

## Abstract

Composite correlated random walks (CCRW) have been posited as a potential replacement for Lévy walks and it has also been suggested that CCRWs have been mistaken for Lévy walks. Here I test an alternative, emerging hypothesis: namely that some organisms approximate Lévy walks as an innate CCRW. It is shown that the tri-modal CCRW found to describe accurately the movement patterns of mussels (*Mytilus edulis*) during spatial pattern formation in mussel beds can be regarded as being the first three levels in a hierarchy of nested movement patterns which if extended indefinitely would correspond to a Lévy walk whose characteristic (power-law) exponent is tuned to nearly minimize the time required to form patterned beds. The mussels realise this Lévy walk to good approximation across a biologically meaningful range of scales. This demonstrates that the CCRW not only describes mussel movement patterns, it explains them.

Lévy walks are a popular model of organism movement pattern data^1^. In a Lévy walk the mean-squared step-length diverges over time and this implies the absence of a characteristic scale and so fractal scaling. It has long been recognised that these superdiffusive and fractal properties of Lévy walks can be advantageous when searching and as a consequence may be selected for^2^,^3^. This expectation is now amply supported by empirical observations. Many organisms including E-coli, T-cells, honeybees, the wandering albatross and some marine predators have been reported to have movement patterns that can be approximated by Lévy walks^4^,^5^,^6^,^7^,^8^,^9^,^10^,^11^,^12^. Nonetheless, Lévy walks have not been accepted in some quarters. This is partly because many earlier studies had wrongly ascribed Lévy walks to some species through the use of inappropriate statistical analyses and through misinterpreting data^13^,^14^. This has cast a long shadow over Lévy walk research^15^,^16^,^17^. It is also because ‘composite correlated random walks' (CCRW) appear to be a strong alternative model of movement pattern data resembling Lévy walks^18^.

In these models, organisms are assumed to switch between two or more kinds of simple walk pattern. CCRW can resemble Lévy walks when frequently occurring movements with relatively short steps are interspersed with more rarely occurring longer steps. This leaves open the question of why CCRW can come to resemble Lévy walks. Close resemblance requires fine tuning of the parameters in a CCRW that determine the step-lengths for each mode and the rates of switching between the different modes. The issue was first articulated by de Jager et al.^19^ who hypothesised that organisms can approximate a Lévy walk by adopting an intrinsic CCRW in which switching between different modes is internally triggered rather than externally triggered, by, for instance, the detection or depletion of food, as in the original model of Benhamou^1^. The hypothesis of de Jager et al.^19^ stemmed from analyses of the movement patterns of mussels (*Mytilus edulis*) made during the formation of regularly patterned beds when individuals are searching for conspecifics^20^. The mussels aggregate with some conspecifics to minimize wave forces from the water, but also keep their distance from other clusters of mussels to avoid high competition. The mussel movement patterns resemble Lévy walks^20^ but are, in fact, better represented by CCRW^21^. This finding led Jansen et al.^21^ to suggest that Lévy walks have been wrongly identified in mussels and to conclude that one has to be cautious in inferring the presence of Lévy walks in biological systems, implying that the concept is not applicable to organisms. Nonetheless, repeated switching between movement strategies induced by changing environmental conditions, as in the model of Benhamou^18^, does not provide a plausible explanation for the observed composite walk, as the mussels were placed in a bare, homogeneous environment^19^. de Jager et al.^19^ also precluded the possibility that variation in individual walking behaviours – for example, multiple different Brownian (exponential) walks - together make up the observed composite walk as Brownian walks fitted individual movement pattern data very poorly. The observed CCRW therefore appears to be intrinsic. de Jager et al.^22^ subsequently showed that the intrinsic pattern is Levy-like in a bare tank (sparse conditions) but emerges as Brownian when encounters with conspecifics are frequent. A similar resemblance between intrinsic CCRW and LW to that seen in the mussels has subsequently been found in the movement patterns of the Australian desert ants *Melophorus bagoti*^23^. In these desert ants a bi-modal walk is utilized when searching in visually unfamiliar surroundings, a setting which favours Lévy walk searching. When searching in visually familiar surroundings, the ants adopt a Brownian walk. The hypothesis of de Jager et al.^19^ finds support in the theoretical analysis of Reynolds^24^ who showed how selection pressures can give intrinsic CCRW Lévy walk characteristics. In this note I show explicitly that the CCRW seen in mussels approximates to a Lévy walk that is optimized for the formulation of patterned beds, i.e., for searching for conspecifics. The approach taken draws heavily upon the work of Hughes et al.^25^ who constructed a family of random walks – now sometimes called Weierstrass random walks or Weierstrass Lévy flights because of their association with the Weierstrass function - having a hierarchy of self-similar clusters that coincides with a Lévy walk. The tri-modal CCRW is shown to correspond to the first three hierarchy levels of a Weierstrass Lévy flight. Weierstrass random walks are one of the simplest random walks which do not satisfy the Central Limit Theorem. In the continuum limit they are governed by Lévy stable distributions and not by Gaussians. Weierstrass random walks have thus become the paradigmatic Markov process giving rise to Lévy walks and have come epitomize scale-invariance^27^. The new finding explains why the CCRW so closely resembles a Lévy walk and accounts naturally for the optimization, as Weierstrass Lévy flights can have similitude with self-avoiding random walks^26^. Self-avoidance is advantageous when randomly searching because it avoids needlessly revisiting previously-searched locations.

## Results

The step-length distribution found to describe accurately the movements of mussels is the tri-exponential 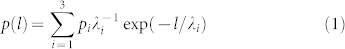
The maximum likelihood estimates for the mode-occurrence probabilities and the mean step-lengths are *p_1_ = 0.867*, *p_2_ = 0.099, p_3_ = 0.034*, *λ_1_ = 0.28 mm*, *λ_2_ = 1.5 mm* and *λ_3_ = 14.5 mm* (reference 21; full data set). Here without loss of generality and to simplify analysis the mean-steps, *λ_i_,* are rescaled so that smallest mean-step has length 1. This gives *λ_1_ = 1.0*, *λ_2_ = 5.4* and *λ_3_ = 51*.

**Figure 1:**
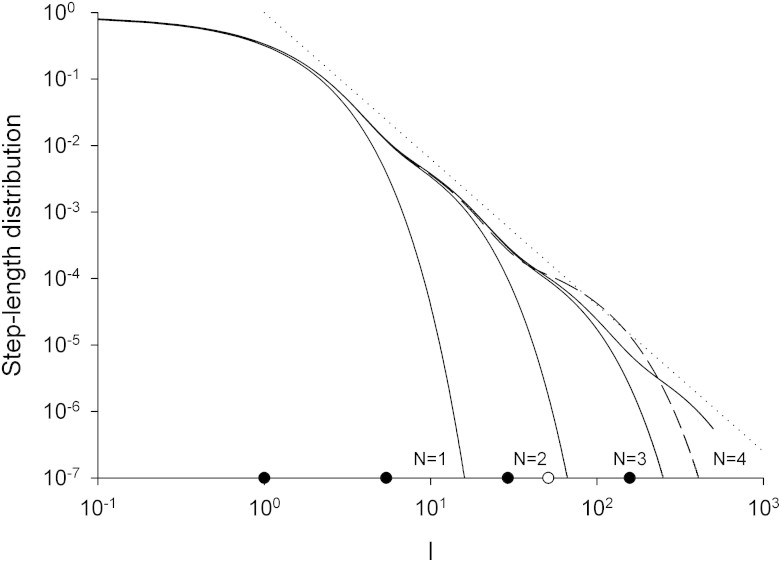
Step-length distributions produced by the progression of Hughes et al.^25^, Eqn. 2, truncated at the *N^th^* hierarchy level. Model parameters values, *q = 7.52* and *b = 5.41*, are derived from the fitting of a tri-exponential by Jansen et al.^21^ to the movement pattern data of de Jager et al.^20^ for mussels (*Mytilus edulis*) during the formation of mussel beds (see text). Step-length distributions are shown for *N = 1*, *2*, *3* and *4* (solid-lines) and for a modified *N = 3* progression that compensates for the absence a *N = 4* level (see text) (dash-line). Shown for comparison (dotted-line) is the *μ* = 2.195 power-law scaling which is obtained from Eqn. 2 when *N* → ∞. The average step-length for the first level has been rescaled to unity. The average step-lengths for each of the uncompensated levels (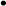) and the compensated 3^rd^ level (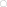) are shown.

**Figure 2:**
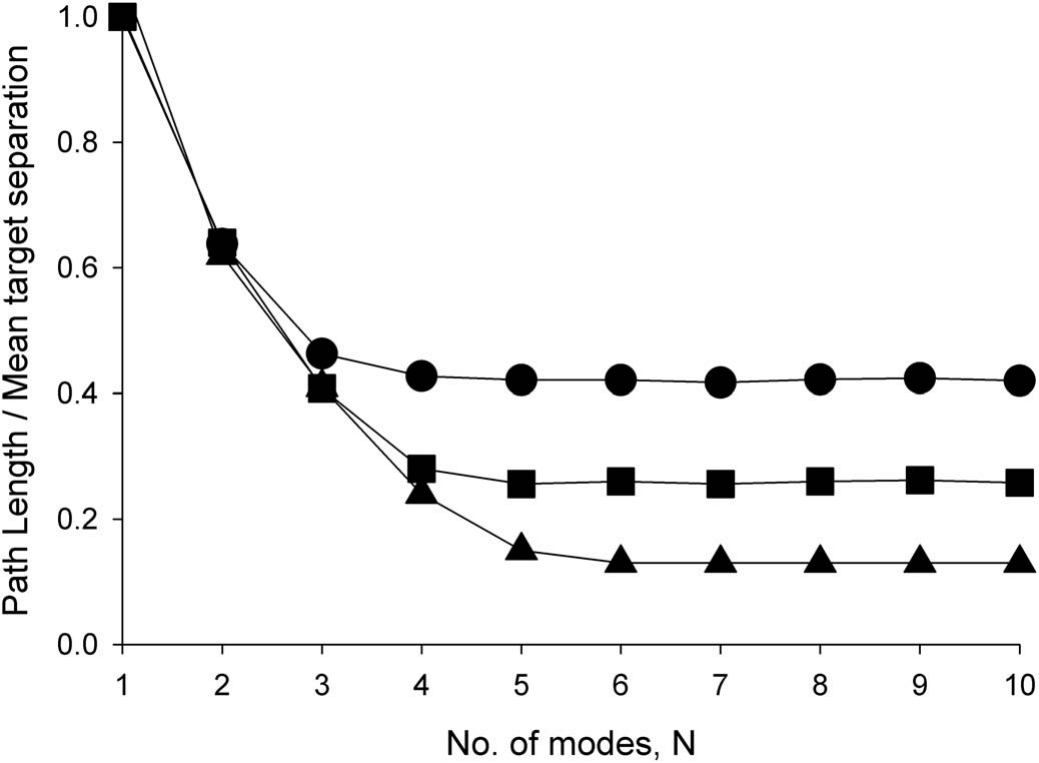
Simulation data showing that the tri-exponential CCRW is as effective as higher-order, hyper-exponential models that better approximate an optimal Lévy search when randomly searching for clusters of conspecifics. The models correspond to the progression of Hughes et al.^25^, Eqn. 2, with *q = 7.52* and *b = 5.41*. The average lengths of the search paths are shown for cluster spacings of 100*λ*_1_(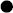), 500*λ*_1_(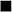) and 5000*λ*_1_ (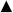). The lines are added to guide the eye.

**Figure 3:**
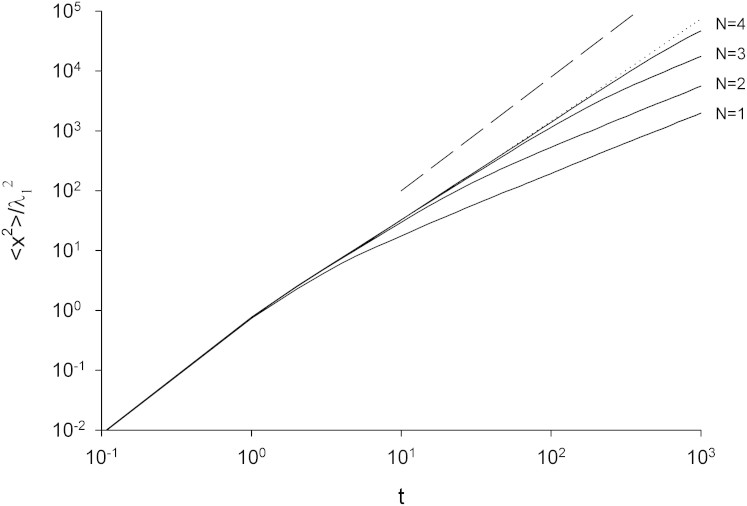
Simulation data for mean-square displacements as functions of time, *t*, for CCRW corresponding to the progression of Hughes et al.^25^, Eqn. 2, *with q = 7.52* and *b = 5.41* and truncated at the *N = 1, 2, 3* and *4* hierarchy levels (solid-lines). Shown for comparison are simulation data for a Lévy walk with *μ* = 2.195 (which is obtained from Eqn. 2 when *N* → ∞) (dotted-line) together with the theoretical expectations, 〈*x*^2^〉 ∝ *t*^4−*μ*^, for a Lévy walk with *μ* > 2 at long-times^32^ (dashed-line). Walkers move with speed *1* in arbitrary speed units.

It is readily seen that the empirical distribution, Eqn. 1, is an approximate, truncated form of the model distribution given by Eqn.2 in the Methods section. The observed occurrence probability for the first mode, *p_1_ = 0.867*, corresponds to *q = 7.53*. With this specification, the occurrence probability of the second mode is predicted to be *0.11*. This prediction differs from observation (*p_2_ = 0.099*) by just 10%. The observed occurrence probability for the third mode is determined by the requirement that the occurrence probabilities sum to unity, and so cannot be meaningfully compared with the model distribution, Eqn. 2, which has many more modes. The observed mean-step lengths *λ_1_ = 1.0* and *λ_2_ = 5.4* correspond to *b = 5.4*. With this specification, the mean-step length in the third mode is predicted to be *b^2^ = 29.1* and so at variance with the observed value *λ_3_ = 51*. Nonetheless, it is possible that the mussels are compensating for the absence of a 4^th^ mode by modifying the step-length in the 3^rd^ mode so that it is equivalent to the arithmetic average step-length of the 3^rd^ and 4^th^ modes. Support for this speculative notion comes from the fact that the predicted value of this average step-length, *b^2^ + b^3^/q = 51*, coincides with the observed value *λ_3_ = 51*. This mismatch between the observed and predicted values of *λ_3_* could also be indicative of the presence of 4^th^ mode that has not been properly resolved in the analysis of Jansen et al.^21^ because there is insufficient data. The occurrence probability for this mode is predicted to be 0.002 which corresponds to just 7 steps in the dataset. The model parameters q = 7.52 and b = 5.41 correspond to a Lévy exponent *μ* = 1+ ln*q*/ln*b* = 2.195. This prediction is close to the observed value, 1.975^21^, (obtained by fitting a power-law to the tail of the step-length distribution using maximum likelihood estimates) and close to the theoretical expectation, *μ* = 2.0, for optimal behaviour^20^. Jansen et al.^21^ obtained a smaller maximum likelihood estimate, *μ* = 1.397, but this was because they had in essence fitted a single power-law to the entire step-length distribution which includes a relatively flat core and a tail. The core could be represented by a power-law with a power-law exponent close to zero. The stepper tail would be better represented by a power-law with a larger power-law exponent. The resemblance between the progression, Eqn. 2, and a step-length distribution with a heavy, *μ* = 2.195 power-law tail is illustrated in Fig. 1. In the presence of 3 or 4 modes, close adherence to a power-law is seen to be attained across about 2 orders from magnitude. The presence of 3 or 4 modes is also significant to attain near optimal search efficiencies when the mean spacing between targets (clusters of conspecifics) is between 100*λ*_1_ and 500*λ*_1_; a range which encompasses empirical observations of mussel beds^20^. The potential to deploy more modes only becomes advantageous when targets are much scarcer (Fig. 2). The close correspondence between the CCRW with 3 or 4 modes and a Lévy walk *per se* with *μ* = 2.195 is further illustrated in Fig. 3 which shows that these models of movement pattern have nearly identical mean-squared displacements characteristics up to the typical time needed to find a conspecific (Fig. 2).

## Discussion

The step-length distribution that characterises mussel movements during patterned bed formation is well represented by a tri-exponential and less well represented by an exponential and by a power-law^21^. Jansen et al.^21^ argued that this convincingly shows that mussels are not doing a Lévy walk but are instead doing a CCRW. Here it was shown that the tri-exponential step-length distribution is finely tuned to produce Lévy walk characteristics of the type predicted to minimize the time for pattern formation^20^. It is common knowledge that long-tailed step-length distributions – the hallmark of Lévy walks - can be approximated by a superposition of several short-tailed distributions. Many such progressions can approximate a Lévy walk, albeit over a limited range of scales. Here it was found that the 3 modes seen in the mussels approximate to the first three terms in the progression proposed by Hughes et al.^25^, i.e., to the first 3 levels of the regular hierarchy structure of a Weierstrassian Lévy walk rather than to other less schematic suppositions that not readily extended to encompass progressively more scales. This identification of the spatial-temporal hierarchical structure in the CCRW addresses directly the pertinent concluding remarks of de Jager et al.^19^ who noted that it is “most advisable to examine the switching behaviour by means of temporal and spatial correlations of movement steps within animal tracks rather than [just] fitting multimodal models to step size distributions”. It is unlikely that such fine tuning of the CCRW has come about by chance. This suggests that the mussels are approximating an optimal Lévy walk by adapting a CCRW, as first hypothesised by de Jager et al.^19^. This is significant because the fundamental mechanisms leading to Levy walks are in general not elucidated and remain the subject of intense study^31^. More generally, it shows that CCRW and Lévy walks are not necessarily competing models of movement pattern data. Instead Lévy walks can be viewed as simple integrative models whilst CCRW with their added complexity provide more mechanistic descriptions of movement patterns. An alternative explanation for the CCRW was posited by Jansen et al.^21^ who suggested that “the smallest average movement (~0.4 mm) is related to non-movement, combined with observational error”; “the next mode (average movement ~ 1.5 mm) is related to mussels moving their shells but not displacing, and the mode with largest movements (on average 14 mm, about the size of a small mussel) is related actual displacement”. This is biologically plausible. Shell-wobble-movements could provide mussels with a way of noting the (continued) presence of neighbouring mussels. The longer displacements could allow mussels to gauge the overall population density or perhaps the size of the entire cluster or distance to other clusters, and thereby facilitate the formation of ‘optimal' clusters. This alternative explanation does not account for the observed frequency of occurrence of the 3 movement modes and so does not explain why the CCRW resembles an optimized Lévy walk.

Although the CCRW does not capture a Lévy walk in its entirety, it does capture its essence; namely a scale-free hierarchy of nested movements across a range of ecologically important scales that allow for intensive searching whilst restricting needless resampling of previously visited location. Such CCRW can, on average, have the same number of subclusters per cluster with a change in scale as self-avoiding random walks, i.e., have the same fractal dimension as random walks that do not visit the same location more than once^26^. Such behaviour has ancient origins and has been seen in the deep-sea trace fossil *Cosmorhaphe* that are typically found in Mesozoic (about 252–66 million years ago) and younger strata^28^. Trace fossils are the preserved form of tracks made by organisms that occupied ancient sea beds. These traces are the only direct record of the behaviour of ancient organisms and thus provide critical indications of the early evolution of movement patterns. Self-avoiding behaviour persists to this today and has, for example, been observed directly in the grazing tails of the Valviferan isopod *Chiridotea coeca* which resides in intertidal zones containing sporadically distributed resources that are replenished semi-diurnally^29^; conditions that can favour Lévy walk search patterns (Viswanathan et al. 1999). Self-avoiding behaviour is also evident in the limpet *Lottia gigantea*^30^. This algal gardener must obtain an adequate ration without compromising the productivity of its garden and achieves this balance by seldom grazing over an area more than once, i.e., the limpets avoid crossing previous grazing trails.

In the case of the mussels, 3 modes are sufficient for Lévy characteristics to be realised across all scales of their searching^20^. The re-interpretation of the analysis of Jansen et al.^21^ posited here therefore brings mussels' movements back into the family of Lévy walks^4^,^5^,^6^,^7^,^8^. For some of these other taxa, as with the mussels, it is possible that the tails of the step-length distributions are strictly not power-laws but are instead better represented by functions with multiple parameters. This, however, masks the resemblance between taxa in the Lévy family, and disguises the apparent ubiquitous occurrence of Lévy-like search patterns.

The correspondence between the theoretical expectations and the empirical data suggests that the movements of ~0.4 mm in mode 1 are not, after all, on a biologically irrelevant scale or a possible artefact of the recording accuracy, as proposed by Jansen et al.^21^. This merits further investigation. A key question is whether mussels do, indeed, have only 3 modes or whether there are, in fact, more modes which are not seen because conspecifics are located and searching ceases prior to their employment. It would therefore be interesting to investigate whether 4^th^ and higher modes in the CCRW are employed by isolated mussels that cannot locate a conspecific. And, if present, whether these modes approximate the 4^th^ and higher hierarchy levels of the Weierstrassian Lévy walk or instead introduce new behaviours, leading to departures from fractal scaling. It would also be interesting to test for the presence of hierarchical CCRW in other taxa.

## Methods

Following Hughes et al.^25^ the step-length distribution is here taken to be a hyper-exponential: 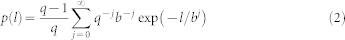
This differs slightly from the original random walk of Hughes et al.^25^ where steps had length *1*, *b*, *b^2^*, *b*^3^, rather than mean lengths *1*, *b*, *b^2^*, *b*^3^ because they were drawn from delta-functions rather than from exponential distributions. This change allows for a connection to be established with CCRW in which step-lengths are typically drawn from hyper-exponential distributions. Notice that a step drawn from an exponential distribution with mean *b^j^* is *q* times more likely than is a step drawn from an exponential with the next longest mean. As a consequence, a walker will typically make a cluster *q* steps with mean *1* before making a step of length *b*, and so initiating a new cluster. About *q* such clusters separated by a distance of about *b* are formed before a step of length *b^2^* is made. And so on. Eventually a hierarchy of clusters within clusters is formed. This is the hallmark of a Lévy walk. Following the analysis of Hughes et al.^25^ it is readily shown that the step-length distribution, Eqn, 2, has infinite variance when *b*^2^ > *q* and in this case corresponds to a Lévy walk with Lévy exponent *μ* = 1 + ln*q*/ln*b*. The construction, Eqn, 2, therefore provides a recipe for approximating Lévy walks as CCRW, an approximation that becomes ever more precise as the number of modes (number of terms included in the summation) increases.

The searching efficiencies of movement patterns resulting from hyper-exponential CCRW corresponding to truncated forms of Eqn. 2 was examined in numerical simulations. These simulations capture some key behaviours of mussels during patterned bed formation but are not intended to be realistic in detail. Mussels stay in places where they can aggregate with direct neighbours, but move away from crowded locations where food becomes limiting, to search for dispersed conspecifics^20^. Each search therefore begins in the neighbourhood of one cluster of conspecifics but distant from all other clusters which because of pattern formation tend to be regularly spaced. In the numerical simulations, clusters of conspecifics are taken to be stationary and regularly spaced, and each search begins at a distance *λ*_1_ from a cluster. A simulated mobile mussel chooses a direction of travel at random (either right or left) and a step-length drawn at random from a truncated form of Eqn. 2. It then moves incrementally towards the new location whilst constantly seeking out conspecifics. If it does not meet a conspecific, it stops after traversing the distance *l* and chooses a new direction of travel and a new step-length. A search ends when the mussel first comes into contact with a conspecific. The average length of a search path was obtained by ensemble averaging over *10^5^* searches.
